# A Green Approach for Recycling Compact Discs

**DOI:** 10.3390/polym15030491

**Published:** 2023-01-17

**Authors:** Francesco Paolo La Mantia, Domenico Liarda, Manuela Ceraulo, Maria Chiara Mistretta

**Affiliations:** 1 Dipartimento di Ingegneria, Università di Palermo, Viale delle Scienze, 90128 Palermo, Italy; 2 INSTM, Consorzio Interuniversitario Nazionale di Scienza e Tecnologia dei Materiali, Via Giusti n. 9, 50121 Florence, Italy

**Keywords:** recycling, compact discs, polycarbonate, sustainable method

## Abstract

Compact discs (CDs) and digital versatile discs (DVDs) are mainly made by polycarbonate disc, a thin layer of aluminum or silver, a thin layer of a coating and a thin layer of a label of paper or PET. The recycling of these discs is difficult due to the removal of these non-polymeric layers and to our best knowledge, no industrial plants have been resent for their recycling. In this work, we propose a facile way to remove the non-polymeric layers and investigate the effect of the repetitive extrusion process on the processability and on the mechanical properties of the recycled polycarbonate. A few works have been published dealing with both the removal of the non-polymeric layers and the mechanical recycling of the disk of polycarbonate. In our approach, the removal of the non-polymeric layers is easily obtained through a thermo-mechanical treatment in a basic solution by ammonia. This process can be considered green because is made at a low temperature with a small amount of water and a very small amount of ammonia, saving energy and water. The properties of the polycarbonate remain good if the mechanical recycling is made after drying the post-consumer polycarbonate.

## 1. Introduction

Compact discs (CDs) and digital versatile discs (DVDs) are made by polycarbonate disc, a thin layer of aluminum or silver, a thin layer of a coating and of a label of paper or PET. This latter layer can be absent if the label is directly printed on the coating. The polycarbonate is more than 95% of the total weight of the disc. Although the CDs are going out of production, many billions of these discs are still in use or unused and, in any case, should be disposed of without any damage to the environment. Moreover, many billions of DVDs are still used and are still produced. It has been evaluated [1] that in 2003, the word production of CDs and DVDs was about 12 billion units. Indeed, while the polycarbonate is a polymer that can be recycled to obtain a good secondary material, the presence of metal, lacquer, paints, paper, PET, etc., prevent an easy and economic way to recover the polycarbonate and other valuable components of these discs. Although several papers have been published on the recycling of CDs [1,2,3,4,5,6,7,8,9,10], only a small number have dealt with both the removal of the non-polymeric layers and the mechanical recycling of the polycarbonate or with the use of polycarbonate as filler [1,2,3,6,7]. In the paper [1], the polycarbonate is separated through the abrasion method and recycled melt. The recycled PC has also been characterized in monopolymer blends with virgin PC. The decrease in some mechanical properties is quite low. In the paper [2], the compact discs were used as a sort of filler in plaster, while in [3] the clean discs were obtained by de-inking from the substrate immersing them in a solution of used wastewater from a tanning factor. The CDs were cleaned by shaking them in wastewater using an ultrasonic cleaning bath at 75 °C for 90 min. The patent [4] describes a technique to remove labels, ink and aluminum in an ultrasonic bath containing a strong aqueous alkaline solution of an alkaline salt or of a base, a chelating additive and a surfactant and agitating the discs with ultrasonic energy for a time sufficient to completely remove the coatings.

The recycling of the polycarbonate and the characterization of the recycled polycarbonate has already been reported in several papers [11,12,13,14,15,16,17]. During the reprocessing, the molecular weight of the polycarbonate decreases because of the thermomechanical degradation undergone during the processing operations at high temperature, even more than 300 °C, but the more relevant degradation occurs only if the polycarbonate is reprocessed without any pre-drying. In this case, the hydrolytic chain scission can give rise to a dramatic decrease in the molecular weight [11,12] and, consequently, of the mechanical properties.

The aim of this work is to report the investigation about the recycling of compact discs by using a green approach in order to remove all the non-plastic components and to recycle the polycarbonate through mechanical recycling. The non-polymeric components have been eliminated from the compact discs by a treatment in aqueous solution with ammonia under ultrasonic treatment. The solution can be easy reused in the same treatment. The presence of the ammonia and higher temperatures strongly improves the kinetics of cleaning, however, the cleaning is possible even in pure water and at room temperature. The power of the ultrasonic treatment is also important. The recycling of the polycarbonate has been simulated by repetitive extrusion steps. The rheological and mechanical results confirm that the polycarbonate maintains good properties only if it is extruded after a careful pre-drying and for a limited number of reprocessing steps. The process of removing the non-polymeric layers here proposed can be considered green because it is made at a low temperature with a small amount of water and very small amount of ammonia. This means a very small amount of energy and water necessary for cleaning the polycarbonate disks, thus saving water and energy.

## 2. Materials and Methods

Different post-consumer compact discs having labels both of paper and PET, immersed in a solution of water and ammonia with different concentration of ammonia, namely 0, 0.25, 0.5 and 0.75% wt/wt, were treated in an ultrasonic cleaner bath DU-32 (Argo Lab) working at a frequency of 40 KHz and with a maximum power of 120 W, for different temperatures, namely 20, 30 and 50 °C, power of the ultrasonic treatment and times. The used ammonia was an aqueous solution with 25% of ammonia (Supelco, Merck, Germany). The value of the pH of the solution is 14. The solutions used for the removal of the layers of the CDs were at 0.25, 0.5 and 0.75% of ammonia.

To reuse the ammonia solutions after the cleaning treatments, the solutions were filtered and recycled adjusting the ammonia concentration to restore the pristine pH, i.e., the pH value of the solution before the treatment, for each ammonia concentration solution.

The clean polycarbonate discs were then subjected to extrusion tests in order to simulate the recycling steps. The extrusion tests were performed in a single-screw extruder Brabender, D = 19 mm, L/D = 25, with a temperature profile of 280–280–285–290 °C; the rotational speed was 100 rpm for all the runs. The extrusion tests were performed on both dry and wet samples in order to verify the effect of the humidity on the possible hydrolytic degradation of the polycarbonate. The polymer was dried at 130 °C for 12 h.

The reprocessed polycarbonate was characterized from a rheological and mechanical point of view.

The Melt Flow Index (MFI) was measured according to ASTM D1238 condition O at T = 300 °C under a weight of 1.2 Kg. The flow curves were obtained by using a rotational rheometer ARES G2 (TA Instruments, New Castle, DE, USA) equipped with a parallel-plate geometry (25 mm diameter), at the test temperature of 280 °C.

Elastic modulus, E, tensile strength, TS, and elongation at break, EB, were obtained in tensile mode using a Instron (USA) mod. 3365 universal machine, at a crosshead speed of 1 mm/min.

## 3. Results

### 3.1. Cleaning of the Compact Discs

**Figure 1:**
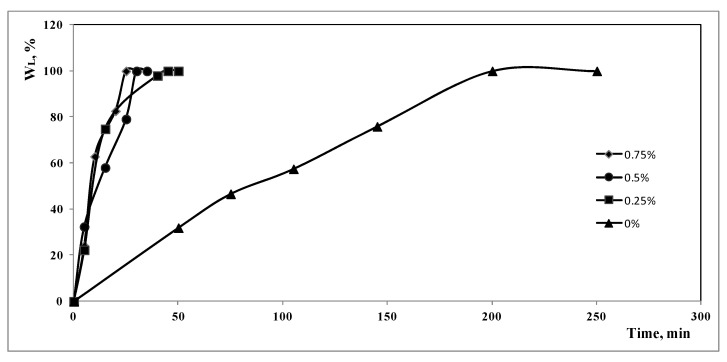
Percentage weight loss during the ultrasonic treatment at T= 30 °C, at the maximum power of the ultrasonic bath and at different ammonia concentrations.

In Figure 1, the weight loss of the CDs during the treatment, calculated as

WL(t) = (W0 − W(t))/(W0 − WF) ∗ 100(1)

is reported as a function of the time of treatment. In Equation (1), WL(t) is the weight loss at a given treatment time, W0 is the initial weight of the CDs, WF is the final weight and W(t) is the weight of the CDs after a given time of treatment. The curves refer to tests carried out at different ammonia concentrations, at the temperature of 30 °C and at the maximum power of ultrasonic bath.

The effect of the concentration of the ammonia is clearly evident: increasing the ammonia concentration, the time necessary to clean the compact disc decreases. It is, however, evident that the cleaning of the CDs is also possible in pure water, but with treatment times much longer. In particular, in pure water the time necessary to clean the CDs, t_f_, is about 200 min, while, in the presence of ammonia, the t_f_ values range from 30 to 50 min, progressing from 0.75 to 0.25% of ammonia. The pH of the solution, reported in Table 1 for the different solutions used, plays an important role in the kinetic of the treatment.

**Figure 2:**
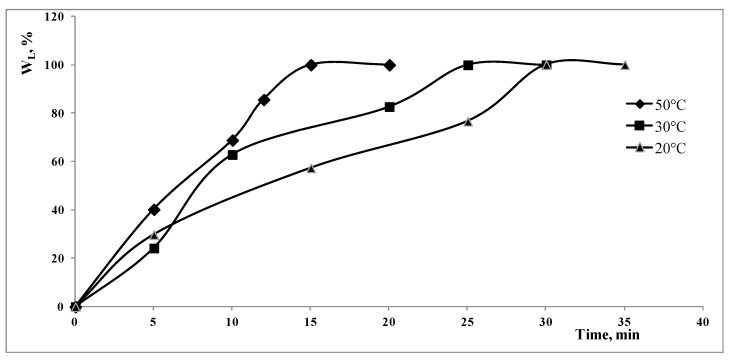
Percentage weight loss during the ultrasonic treatment at the ammonia concentration of 0.75%, maximum power of the ultrasonic bath and at three different temperatures.

In Figure 2, the percentage weight loss during the treatment is reported as a function of the treatment time at different temperatures, at the maximum power of the ultrasonic bath and at an ammonia concentration of 0.75%.

The temperature has a remarkable effect on the time necessary to clean the CDs and increasing the temperature from 30 to 50 °C, the value of t_f_ becomes about one half, progressing from 30 to 15 min.

**Figure 3:**
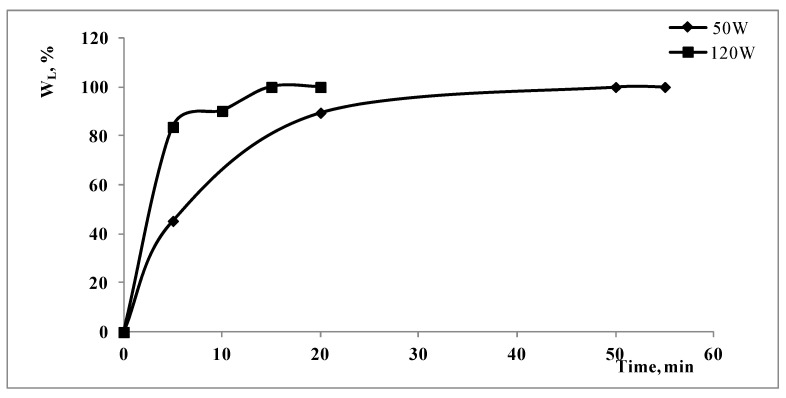
Percentage weight loss during the ultrasonic treatment at the ammonia concentration of 0.75%, at the temperature of 50 °C and at two different levels of power of the ultrasonic bath.

In Figure 3, the percentage weight loss to remove the non-polymeric components is reported as a function of the treatment time, at the ammonia concentration of 0.75%, at the temperature of 50 °C and at two different values of the power of the ultrasonic cleaner bath, namely, the maximum value of 120 W, and at about one half of the maximum power of 50 W.

The power of the ultrasonic cleaner bath, and then the mechanical stress transmitted to the solution and to the compact discs, plays a very important role in the treatment because when increasing the power from 50 W to 120 W, the value of t_f_ becomes less than one half, progressing from about 50 to about 20 min.

**Figure 4:**
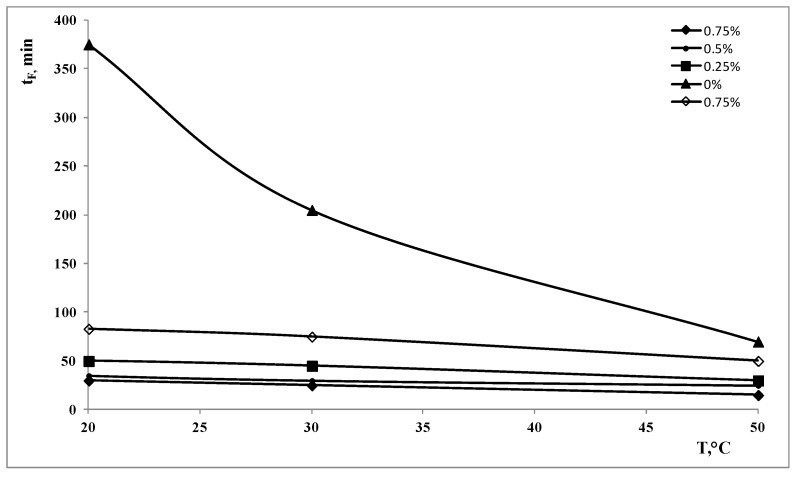
Time necessary to completely remove all the non-polymeric components, t_f_, at the two values of the power as a function of the treatment temperature and different NH_3_ concentration. Full symbols: 120 W, empty symbols: 50 W.

In Figure 4, the time necessary to completely remove all the non-polymeric components, t_f_, in all the treatment conditions have been reported as a function of temperature and at different ammonia concentrations for the two values of the power of the ultrasonic bath.

From this figure, it is clearly evident that the effect of the ammonia concentration is remarkable only at the lower temperatures, while, at the higher temperatures, the value of t_f_ is certainly less dependent on the ammonia concentration. The same comments can be made for the effect of the power of the ultrasonic bath: the effect of the ammonia concentration is less important with increasing the mechanical power.

**Figure 5:**
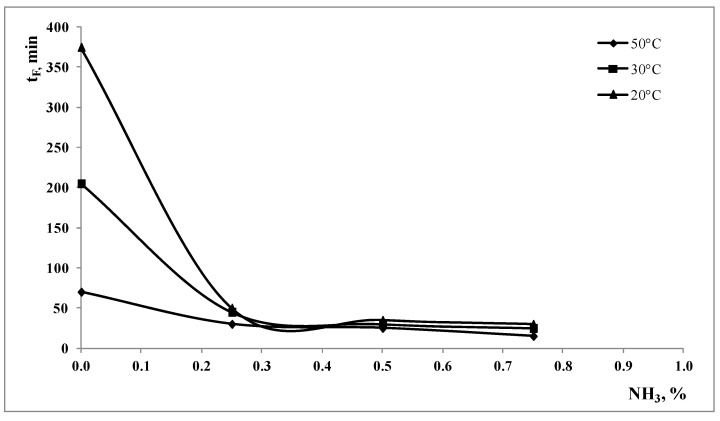
Time necessary to completely remove all the non-polymeric components, t_f_, at the maximum power as a function of the ammonia concentration and different temperatures.

In Figure 5, the values of t_f_ are reported as a function of the ammonia concentration for the three values of the temperatures and for the values of the power.

In this case also, the effect of the temperature is relevant only at low values of the ammonia concentration and at low value of the power, while, with increasing temperature and power, the effect of the ammonia concentration is less relevant.

All the experimental data clearly suggest that temperature and mechanical stress are the two more important driving forces, while the ammonia concentration plays a remarkable role only at low temperature and low mechanical stress. The treatment is mainly a thermomechanical pulping where the role of the pH of the alkaline solution is to improve the kinetic of the treatment.

**Figure 6:**
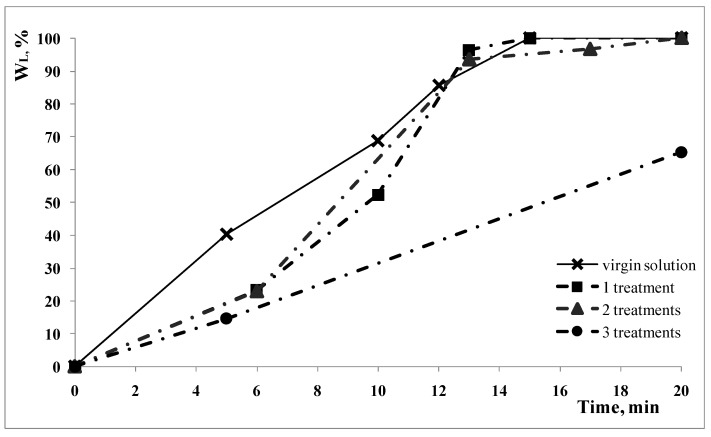
Percentage weight loss as a function of time for cleaning treatment with recycled solutions.

This effect is clearly evidenced when the solution used for the ultrasonic treatment is the same solution recycled after one or more treatments. In Figure 6, the weight loss as a function of the time at fixed temperature and power of the ultrasonic bath is reported as a function of the time when the solution is a virgin solution at 0.75% of ammonia concentration and the same solution after one, two and three treatments where the complete cleaning of the CDs was obtained. The tests were carried out at *t* = 50 °C and at the maximum power of the ultrasonic bath.

The curves are evidence as to how the treatment time at which the CDs are cleaned increases with increasing the number of recycling. In Table 2, the values of the pH of these solutions are reported.

The value of the pH slightly decreases with the number of treatments, the time necessary to completely remove the non-polymeric components increases.

### 3.2. Reprocessing of the Polycarbonate

**Figure 7:**
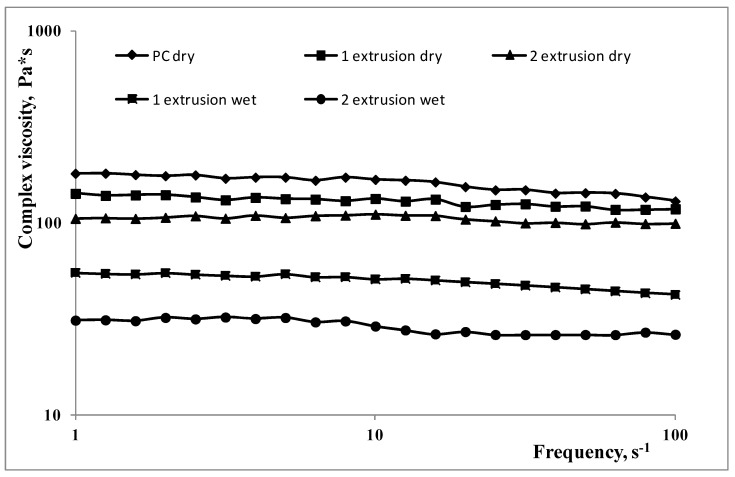
Flow curves of the post-consumer polycarbonate after the extrusion steps for both dry and wet samples.

The flow curves of the polycarbonate samples are reported in Figure 7 for the dry and wet samples. With increasing the reprocessing steps, the viscosity of the polycarbonate decreases. This is due to the thermomechanical degradation for the dry samples and to the thermomechanical degradation and, mainly, to the hydrolytic degradation for the wet sample.

**Figure 8:**
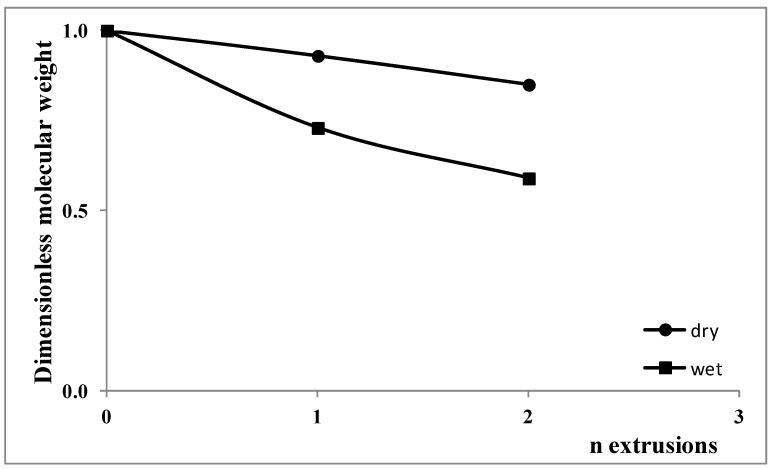
Dimensionless molecular weight.

In Figure 8, the dimensionless decreases in the molecular weight as a function of the processing steps for both samples are reported.

The dimensionless decrease in the molecular weight was calculated following the equation:

η_0_ = k M_W_^3.4^ (2)

The molecular weight of the polycarbonate decreases with increasing the number of processing steps and progressing from dry to wet conditions. The decrease in molecular weight after one extrusion in wet conditions is, however, lower than that measured after two extrusions in dry conditions.

**Figure 9:**
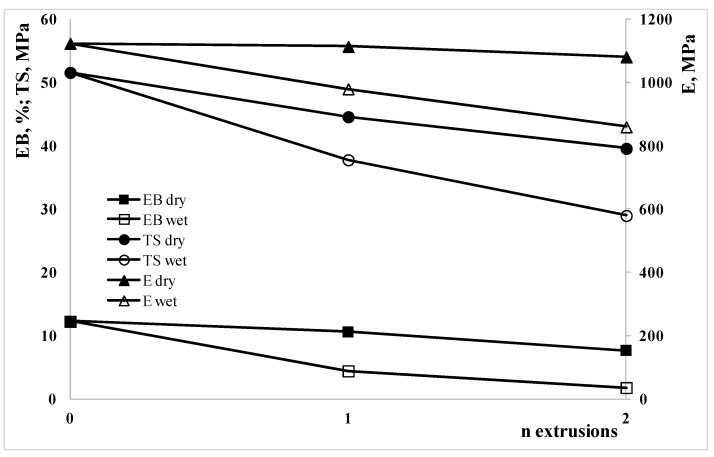
Elastic modulus, TS, tensile strength, and, EB, elongation at break as a function of the extrusion steps, for both dry and wet samples.

The mechanical properties, E, elastic modulus, TS, tensile strength, and, EB, elongation at break are reported in Figure 9, as a function of the extrusion steps, for both dry and wet sample.

The mechanical properties in an amorphous polymer such as polycarbonate depends mainly on the molecular weight. Indeed, the decrease in all the mechanical properties follow the decrease in the molecular weight. The mechanical properties of the sample extruded two times in dry conditions are much better of the sample extruded only one time but in wet conditions confirming that the drying step is a necessary step for the recycling of the polycarbonate.

## 4. Conclusions

The first step for the recycling of compact discs and DVDs is to remove the label and the layers’ coating. In this work it has been demonstrated that a treatment in an aqueous solution of ammonia in an ultrasonic bath is able to completely remove both labels and coating. The time necessary to clean the CDs strongly decreases with increasing the temperature, the power of the ultrasonic bath and the pH of the solution. However, the cleaning of the CDs is also possible only with water but with longer times. However, with increasing the temperature, the effect of the basicity of the solution becomes less important and the cleaning also occurs with pure water in short times. This approach can be considered a green method because it only needs small amounts of water and energy and, in order to shorten the processing time, only a small content of ammonia is necessary.

The polycarbonate has been extruded up two times in dry and wet conditions. It has been shown that the properties of the sample remain similar to the virgin material only if the polymer has been pre-dried. The hydrolytic degradation causes a decrease in the molecular weight much more relevant that the thermo-mechanical degradation during extrusions.

## Figures and Tables

**Table 1:** pH values of the solutions.

Solution	pH
Distilled water, 0%	7
Ammonia 0.25%	11.7
Ammonia 0.5%	11.9
Ammonia 0.75%	12.1

**Table 2:** Values of the pH for different solutions.

Solution	pH
Virgin, 0.75%	12.1
After 1 treatment	11.21
After 2 treatments	10.41
After 3 treatments	9.06

## Data Availability

The data presented in this study are available on request from the corresponding author.
